# The effect of L-Carnitine supplementation on clinical symptoms, C-reactive protein and malondialdehyde in obese women with knee osteoarthritis: a double blind randomized controlled trial

**DOI:** 10.1186/s12891-021-04059-1

**Published:** 2021-02-17

**Authors:** Farnaz Baghban, Mahdieh Hosseinzadeh, Hassan Mozaffari-Khosravi, Ali Dehghan, Hossein Fallahzadeh

**Affiliations:** 1 Nutrition and Food Security Research Center, Shahid Sadoughi University of Medical Sciences, Yazd, Iran; 2 Department of Nutrition, School of Public Health, Shahid Sadoughi University of Medical Sciences, Yazd, Iran; 3 Rheumatology, Department of Internal Medicine, Shahid Sadoughi Hospital, Shahid Sadoughi University of Medical Sciences, Yazd, Iran; 4 Department of Biostatistics and Epidemiology, Research Center of Prevention and Epidemiology of Non-Communicable Disease, Faculty of Health, Shahid Sadoughi University of Medical Sciences, Yazd, Iran

**Keywords:** L-carnitine, Osteoarthritis, CRP, MDA

## Abstract

**Backgrounds:**

L-carnitine decreases oxidation and inflammation by reducing the fatty acid in plasma and using oxygen in ATP synthesis. As such, knee osteoarthritis (KOA) can be improved by reducing apoptotic chondrocytes. This study was designed to compare the effect of L-carnitine supplementation and low-calorie diet on improving KOA among obese women. We further investigated the effect of L- carnitine on improvement of KOA in obese women on low calorie diet.

**Methods:**

To conduct the study, 76 obese women with KOA were randomly assigned into two low-calorie diet groups: the first received 1000 mg of LCG and the second took the placebo (PLG) (*n* = 38). Anthropometry indices, body composition, lipid profile, C-reactive Protein (CRP), Malondialdehyde (MDA), and the Western Ontario and McMaster Universities Arthritis Index (WOMAC) were assessed at the baseline condition and after 12 weeks.

**Results:**

The mean change of body mass index (BMI) (− 1.21 ± 0.84 vs. -0.79 ± 0.70; *P* = 0.02) and weight (− 2.76 ± 1.69 vs. -1.95 ± 1.73; *P* = 0.05) were significant in the LCG compared with the PLG. Likewise, LCG compared to the PLG showed insignificant improvement in waist circumference (WC) (− 5.65 ± 5.85 vs. -3.64 ± 3.37; *P* = 0.08). Total cholesterol (*P* = 0.02), MDA (*P* = 0.03), fat mass (P = 0.03) and visceral fat (*P* = 0.001) only showed decreased levels in LCG in comparison to the baseline condition. There was no significant difference between LCG and PLG, in the mean changes of hip circumference, visceral fat, free fat mass, fat mass, lipid profiles, CRP, MDA as well as stiffness, physical function, decrease of pain and total scores (*P* > 0.05).

**Conclusion:**

The 12-week L-carnitine supplementation could improve BMI, but had no significant effect on other anthropometric and body composition measures as well as clinical symptoms, CRP, MDA, and lipid profile in women with KOA. Further trials with higher doses and longer durations are required. IRCT registration number: IRCT2017011932026N2. Registration date: 2017-04-27.

## Background

Osteoarthritis (OA), as one of the most common type of arthritis, is recognized as a progressive degenerative joint disease [1]. Knee osteoarthritis (KOA) causes chronic pain, disability, and morbidity which consequently impose enormous burden on global health and social care systems [2]. Age, obesity, gender (women), and genetic are the most important contributors to the development of KOA [3]. Oxidative stress is associated with pathogenesis of OA. In OA, elevated levels of reactive oxygen species (ROS) and lipid peroxidation products such as oxidized low-density lipoprotein (ox-LDL) and malondialdehyde (MDA) in chondrocytes induce pain and physical disability [4]. Also, plasma and synovial fluid level of MDA are further observed in patients with OA [5]. Recent studies have demonstrated that local inflammation plays a critical role in the development and progression of OA [6]. Circulating C-reactive protein (CRP) as a systemic biomarker of inflammation indicates increased levels in patients with OA [7].

L-carnitine (4-N-trimethylammonium-3-hydroxybutyric acid) can remarkably transfer the long-chain fatty acids from the inner mitochondrial membrane to the peripheral tissues [8]. It reduces the plasma free fatty acids, uses oxygen for ATP synthesis, and decreases oxidation and inflammation [9]. Thus, KOA can be improved by reducing apoptotic chondrocytes in OA cartilage [10]. Some studies also showed carnitine concentration reduction in blood and tissues of patients with rheumatoid arthritis [11]. L-carnitine intake can reduce KOA symptoms and serum matrix metallopeptidase13 (MMP13) in rats with OA [12]. Earlier studies further showed that L-carnitine significantly reduced CRP and MDA in healthy adults as well as patients with coronary arthritis disease and hemodialysis [13–15]. A meta-analysis also revealed that L-carnitine supplementation significantly reduced serum levels of total cholesterol and low-density lipoprotein cholesterol (LDL-c) in diabetic patients [16]. However, the results of a few other studies witnessed that 750 mg/d L-carnitine supplementation had no effect on serum lipid profile and CRP in women with KOA [17, 18]. Another study on hemodialysis patients reported that 12-week L-carnitine supplementation improved CRP, but had no significant effect on oxidative stress [19].

According to the above evidences, the findings of studies investigating the effect of L-carnitine on lipid profile, CRP, and some other oxidative indices seem controversial. Also, the effect of this supplementation on KOA is still poorly understood. As such, the first objective of this study was to evaluate the effect of oral L-carnitine supplementation on CRP, MDA, lipid profile, Western Ontario and McMaster Universities Arthritis Index (WOMAC), as well as anthropometry and body composition measures in obese women with KOA.

The literatures also confirm that low calorie diet had a therapeutic effect on KOA [20, 21]. Previous studies were conducted on the effect of L-carnitine supplementation without calorie restriction. The aim of the present study was to compare the effect of L-carnitine supplementation and low-calorie diet on improvement of KOA among obese women. We aimed to investigate the effect of L-carnitine supplementation on improvement of KOA in obese women on low calorie diet.

## Methods

### Study cases

The clinical trial was conducted according to the CONSORT guidelines where 100 women with KOA were recruited from the Khatam Al-Anbia Clinic of Rheumatology Department in Yazd, Iran. The selection criteria complied with age ≥ 45 years, body mass index (BMI) in the range of 25–35 kg/m^2^, and diagnosis of KOA according to the clinical classification of KOA [22]. Cases with former or planned knee-joint replacement, being under pharmacologic treatment for obesity, having no history of or active rheumatic diseases, using no nonsteroidal anti-inflammatory drug (NSAID), consuming multivitamin, minerals or other nutritional supplements, and having severe liver, kidney, or heart diseases were excluded from the study. Moreover, those cases taken less than 80% of the prescribed L-carnitine and placebo tablets were also excluded.

### Randomization and intervention

The present study was a double blind randomized controlled trial. Patients with the aforementioned criteria were divided into the L-carnitine group (LCG) and placebo group (PLG) through randomization lists made by a computerized random-number generator and simple randomization process with the ratio of 1:1. The LCG received 1 g/d L-carnitine and the PLG received 1 g/d placebo for 12 weeks. The placebo pills contained inactive ingredients with no therapeutic activity and had an identical appearance. All tablets were produced by Karen Pharmaceutical & Nutrilife Co., Yazd, Iran. As a double-blind study, the placebo and supplement bottles were labeled as A and B, respectively, by the factory, but neither the patients nor the research team members were aware of the codes. Every month, patients received a bottle of tablet containing 30 tablets. Compliance rate was monitored by the research personnel using pill counts and patients’ self-reporting. Participants who did not consume more than 20% of their supplements were eliminated from the analysis. All participants followed a low-calorie diet. A registered dietitian estimated the energy expenditure of each patient through Harris-Benedict formula using the individual activity factor [23]. The recommended composition of the diet was 50 to 60% carbohydrates, 15 to 20% proteins, and less than 30% total fat. A dietitian completed the 3-day food recall for all participants at the baseline of the intervention and visited patients every month to check their compliance with the diet according to the patients’ feedback and 24-h food recall. At the baseline, physical activity during the past week was assessed using the long version of International Physical Activity Questionnaire (IPAQ). Patients were also prohibited from changing their level of activity during the study.

### Outcome measurements

The following measures were assessed at the baseline condition as well as 12 weeks after initiating the treatment: primary outcome included WOMAC, CRP, and MDA. Secondary outcome was LDL-c, TC, high density lipoprotein-cholesterol (HDL-c), triglycerides (TG), BMI, fat mass, free fat mass, as well as waist circumference (WC) and hip circumference (HC).

To conduct the laboratory tests, 5 mL of venous blood samples was obtained after the patients had fasted for 8 h overnight. Serum samples were produced from the collected blood samples immediately after the centrifugation (3000 g, 10 min). They were then frozen at − 20 °C, stored at − 70 °C, and measured at the same time. The total TC, HDL-c, and TG were later measured using Pars Ammon kit (Iran). LDL-c was then calculated using Friedewald’s equation [24]. Serum CRP and MDA concentrations were measured through enzyme-linked immunosorbent assay kits and thiobarbituric acid reactive substances Zellbio kit (Germany), respectively.

To assess the clinical symptoms, WOMAC questionnaire was employed. Patients filled out the Persian version of WOMAC index [25] which consists of 24 questions (related to pain, stiffness, and physical function). Items were answered on a Likert scale: none (0), mild (1), moderate (2), severe (3), or extreme (4).

The weight and body composition were also measured for all patients using a portable digital scale (Omeron BF511, Japan) with an accuracy of 100 g. Participants were in light clothes and stood on the scale without help. Furthermore, the height was measured in standing position without shoes using an audiometer fixed on a straight wall to the nearest 0.1 cm. Measuring WC was performed to the nearest 1 cm using non-stretch plastic tape placed midway between iliac crest and lowest rib while participants were in standing position. Moreover, HC was measured over the largest part of buttocks with the accuracy of 1 cm. BMI was also calculated as weight (kg) divided by height squared (m^2^). Fat mass, and free fat mass were also measured for all patients using a portable digital scale (Omeron BF511, Japan).

For physical activity, the Persian version of IPAQ was applied [26]. The continuous score shows the weekly energy expenditure expressed in MET-min/week (metabolic equivalent-minutes). Individuals were classified into three categories of ‘inactive’, ‘moderately active’, and ‘highly active’ using the categorical classification.

### Sample size

Power calculations were conducted based on the pain scores of 72women with KOA who participated in the trial of Kolahi et al. [27]. Assuming 10% dropout rate, we estimated that a total sample size of 76 patients (38 patients per group) would provide 80% of the power to detect a 2.6 pain score difference between PLG and LCG.

### Statistical analysis

Statistical analyses were carried out using SPSS (version 16). The normal distribution of variables was tested by the Kolmogorov-Smirnov test. Differences in patients’ anthropometrics, WOMAC scores, and hematological measurement data between PLG and LCG were analyzed by the Student’s t-test or the Mann–Whitney rank sum test for parametric and non-parametric continuous variables, respectively. The paired t-test or Wilcoxon signed rank test was used to analyze the data within each group before (baseline) and after the intervention (week 12). The analysis of covariance (ANCOVA) was used to identify the differences between the two groups after adjusting for the change in weight. Results were considered statistically significant at *P* < 0.05. Normal data were indicated by means ± standard deviations (SD) and the non-normal scores were presented with median and inter-quartile range (IQR). The dietary information was analyzed with the N4 software (Nutritionist: version 4.0; Tinuviel Software, Warrington, United Kingdom).

### Efficacy and tolerability assessment

For the safety, all participants were interviewed every month for any signs of L-carnitine toxicity or diet-related adverse problems, including serious illnesses or hospitalizations.

## Results

### Characteristics of study participants

**Fig. 1:**
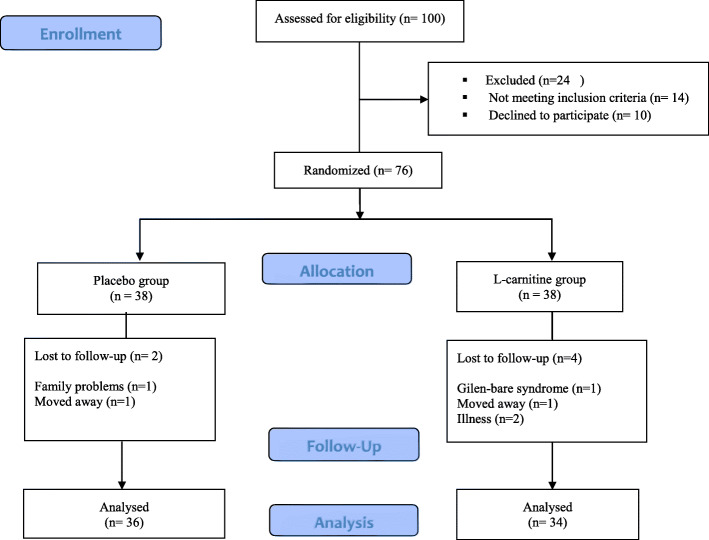
Participant flowchart showing numbers of participants who were recruited, were randomly assigned, dropped out, and were analyzed during the trial

The sampling and trial profiles are summarized in Fig. 1. The baseline characteristics of these patients are shown in Table 1. Participants included 76 women with a mean age of 54.73 ± 7.41, BMI of 32.65 ± 5.60 kg/m^2^, and the body fat percentage of 44.42 ± 5.90%. The median baseline of CRP was 3.52 ± 4.33 mg/dL. Anthropometric parameters, lipid profile, CRP, physical activity, and diet composition such as total energy, protein, fat, and carbohydrate intake, as well as education and occupational status did not differ significantly (*P* > 0.05) between the groups at the baseline condition.

**Table 1:** Selected baseline characteristics of study participants

	L-carnitine group (***n*** = 34)	Placebo group (***n*** = 36)	P^**a**^
**Age (y)**	55.01 ± 7.12	54.43 ± 7.80	0.737
**BMI (kg/m2)**	33.03 ± 6.67	32.10 ± 4.29	0.471
**Height(m)**	1.55 ± 0.10	1.56 ± 0.55	0.481
**Weight (Kg)**	78.69 ± 10.86	78.90 ± 12.17	0.937
**Waist circumference (Cm)**	105.13 ± 9.04	105.92 ± 10.57	0.728
**Hip circumference (Cm)**	115.63 ± 9.37	117.87 ± 10.56	0.332
**Physical activity**	548.01 ± 1131.4	540.01 ± 620.25	0.432
**Energy (kcal)**	1426.21 ± 344.91	1377.72 ± 406.44	0.577
**Carbohydrate (g)**	174.76 ± 37.21	177.38 ± 66.75	0.834
**Fat (g)**	60.83 ± 24.77	56.19 ± 27.55	0.443
**Protein (g)**	50.52 ± 19.34^b^	50.63 ± 22.17^b^	0.455
**Education**			
Illiterate	3 (7.9)	1 (2.6)	0.29
Elementary school graduate	18 (47.4)	20 (52.6)	
Middle and high school graduate	13 (34.2)	14 (36.8)	
University graduate	4 (10.5)	3 (7.9)	
**Occupational status**			0.42
housewife	30 (78.9)	34 (89.5)	
employee	3 (13.9)	2 (5.3)	
Retired	5 (7.2)	2 (5.3)	

Mean ± SD (all such values except protein)

^a^Determined with the use of independent samples t tests for differences at baseline between L-carnitine and placebo groups

^b^Median and IQR (for no normally distributed variables)

### Blood lipids and lipoproteins, CRP and MDA

No significant decrease was observed in the LDL-c, HDL-c, TG, and CRP concentrations in either the LCG or PLG as compared with the baseline. The findings showed that the LCG had lower TC (LCG: *P* = 0.021; PLG: *P* = 0.25) and MDA (LCG: *P* = 0.035; PLG: *P* = 0.36) in comparison to the PLG. However, no significant difference was observed between the LCG and the PLG in terms of the mean changes of lipid profiles, CRP, and MDA concentrations (Table 2).

**Table 2:** Lipids, CRP concentration and WOMAC score changes in patients treated with placebo and L-carnitine before and after 12 weeks of treatment

		L-carnitine group (***n*** = 34)	P^b^	Placebo group (***n*** = 36)	P^c^	P^d^
**LDL cholesterol (mg/dL)**	before	136.13 ± 43.71	0.240	126.92 ± 35.33	0.380	0.316
	after	130.97 ± 29.46		122.94 ± 44.18		0.372
	change	−7.68 ± 37.07		−3.05 ± 20.59		0.525
**HDL cholesterol (mg/dL)**	before	64.97 ± 26.35	0.434	64.08 ± 19.60	0.413	0.867
	after	62.26 ± 11.35		67.94 ± 15.24		0.083
	change	−3.85 ± 8.35		2.5 ± 18.12		0.265
**Triglycerides (mg/dL)**	before	185.53 ± 92.50	0.23	193.63 ± 80.05	0.888	0.684
	after	169.15 ± 73.81		193.44 ± 78.60		0.188
	change	−20.50 ± 97.72		1.50 ± 63.18		0.265
**Total cholesterol (mg/dL)**	before	226.74 ± 50.55	0.021	220.71 ± 42.70	0.259	0576
	after	212.24 ± 41.39		210.89 ± 61.00		0.914
	change	−18.47 ± 44.33		−8.94 ± 46.77		0.385
**CRP (mg/dL)**	before	3.40 ± 2.51^b^	0.142	3.9 ± 3.92 ^a^	0.682	0.618
	after	3.00 ± 3.05		4.40 ± 5.90		0.969
	change	−0.6 ± 2.52		0.09 ± 3.72		0.383
**MDA (mg/dL)**	Before	33.3 ± 36.09	0.03	24.2 ± 14.7	0.36	0.36
	After	26.1 ± 22.22		21.03 ± 11.4		0.76
	change	−5.22 ± 24.98		−2.08 ± 13.5		0.22
**Pain index**	before	7.34 ± 3.4	0.001	8.63 ± 3.63	0.001	0.322
	after	4.71 ± 2.65		5.89 ± 3.54		0.119
	change	−2.91 ± 2.03		−2.89 ± 2.21		0.964
**Stiffness**	before	2.28 ± 1.72	0.001	3.02 ± 2.18	0.001	0.107
	after	1.56 ± 1.50		2.00 ± 1.74		0.261
	change	−1 ± 1.00		−1.11 ± 1.43		0.223
**Physical function**	before	17.82 ± 7.75	0.001	20.63 ± 8.77	0.001	0.142
	after	11.15 ± 6.56		15.61 ± 8.17		0.014
	change	−6.97 ± 3.95		−5.11 ± 5.29		0.102
**Global Score**	before	27.45 ± 11.44	0.001	32.29 ± 12.93	0.001	0.088
	after	17.41 ± 9.81		23.50 ± 12.02		0.024
	change	−10.59 ± 5.58		−9.11 ± 6.44		0.342

Mean ± SD (all such values)

^a^Median; IQR (all such values for non-normally distributed variables)

^b^Determined with the use of paired Student’s t tests for differences between baseline and follow-up in the L-carnitine group

^c^Determined with the use of paired Student’s t tests for differences between baseline and follow-up in the placebo group

^d^Determined with the use of independent samples t tests between L-carnitine and placebo groups

### WOMAC index

Compared with the baseline results, decrease of pain, stiffness, physical function, and total scores were significant in both groups after 12 weeks of treatment (*P* = 0.001). Significant difference (*P* = 0.014) was also found in physical function between the LCG with a mean of 11.15 ± 6.56 and PLG with a mean of 15.6 ± 8.2. Furthermore, at the week 12, the LCG patients had significantly lower total scores (17.41 ± 9.81 vs, 23.50 ± 12.02) than those in the PLG (*P* = 0.024). However, there was no significant difference between the LCG and the PLG in the terms of stiffness and decrease of pain at the end of the study period. No significant difference was found between the LCG and the PLG regarding the mean changes of stiffness, physical function, decrease of pain, and total scores (Table 2).

### Anthropometry and body composition

The weight, BMI, as well as the WC and HC decreased significantly in both groups after 12 weeks of intervention (*P* = 0.001) as compared with the baseline condition (Table 3). Furthermore, a significant difference in terms of visceral fat (P = 0.001) and fat mass (*P* = 0.03) was observed in the LCG at the end of the study. The mean change of BMI (mean changes: − 1.21 ± 0.84 vs. -0.79 ± 0.70; *P* = 0.02) and weight (mean changes: − 2.76 ± 1.69 vs. -1.95 ± 1.73; *P* = 0.05) were significant in the LCG compared with the PLG. The LCG compared to the PLG did not show significant improvement in WC (mean changes: − 5.65 ± 5.85 vs. -3.64 ± 3.37; *P* = 0.088). No significant difference was found between the LCG and PLG regarding the mean changes of HC, visceral fat, free fat mass, and fat mass (*P* > 0.05).

**Table 3:** Anthropometry changes in patients treated with placebo and L-carnitine before and after 12 weeks of treatment

		L-carnitine group (***n*** = 34)	P^**a**^	Placebo group (***n*** = 36)	P^**b**^	P^**c**^
**Weight (Kg)**	before	78.7 ± 10.86	0.001	78.91 ± 12.18	0.001	0.937
	after	75.19 ± 10.84		76.99 ± 12.70		0.527
	chnge	−2.76 ± 1.69		−1.95 ± 1.73		0.052
**BMI (kg/m2)**	before	33.04 ± 6.67	0.001	32.10 ± 4.30	0.001	0.471
	after	31.87 ± 6.56		31.29 ± 4.56		0.669
	change	−1.21 ± 0.84		−0.79 ± 0.70		0.027
**Waist circumference (Cm)**	before	105.13 ± 9.04	0.001	105.92 ± 10.57	0.001	0.728
	after	99.45 ± 11.72		102.39 ± 10.1		0.262
	change	−5.65 ± 5.85		−3.64 ± 3.37		0.081
**Hip circumference (Cm)**	before	115.63 ± 9.37	0.001	117.87 ± 10.56	0.001	0.332
	after	108.44 ± 9.67		112.19 ± 10.58		0.127
	change	−6.82 ± 3.56		−5.64 ± 4.07		0.201
**Free fat mass (%)**	before	24.00 ± 1.74	0.8	24.59 ± 2.64	0.531	0.253
	after	24.11 ± 1.75		23.99 ± 3.78		0.865
	change	0.04 ± 0.09		−0.71 ± 3.47		0.384
**Fat mass (%)**	before	45.19 ± 4.85	0.03	43.63 ± 6.77	0.915	0.251
	after	44.07 ± 4.33		43.95 ± 5.66		0.921
	change	−0.71 ± 1.83		−0.3 ± 1.93		0.360
**Visceral fat (%)**	before	10.71 ± 1.95	0.001	10.47 ± 1.82	0.644	0.587
	after	10.24 ± 2.104		10.50 ± 1.89		0.581
	change	−0.41 ± 0.49		−0.55 ± 0.752		0.320

Mean ± SD (all such values)

^a^Determined with the use of paired Student’s t tests for differences between baseline and follow-up in the L-carnitine group

^b^Determined with the use of paired Student’s t tests for differences between baseline and follow-up in the placebo group

^c^Determined with the use of independent samples t tests between L-carnitine and placebo groups

### Tolerability

Both L-carnitine and placebo were tolerated well in all patients. In the LCG, one patient complained of skin dryness and two complained of slight stomachache. In the PLG, two patients complained of skin dryness and three complained of stomachache**.**

## Discussion

This 12-week randomized placebo-controlled trial examined the effect of oral l-carnitine (1000 mg/d) supplementation compared with placebo in obese women with KOA, who received a low-calorie diet. A significant improvement was observed in BMI while no significant improvement was observed in weight and WC. Also, other anthropometric parameters, lipid profile, CRP, MDA, and WOMAC score did not change significantly.

To the best of our knowledge, this is the first study investigating the effect of L-carnitine supplementation on improving KOA in obese women receiving weight loss diet.

Obesity is important risk factors in the pathogenesis of KOA. Former studies confirmed that weight loss can alleviate pain and improve physical function [20, 21]. Therefore, it seems that both L-carnitine supplementation and weight loss diet are beneficial for OA.

The present study showed a significant decrease in BMI, but other anthropometric variables revealed no significant changes. A few other studies assessing the effect of L-carnitine on other diseases did not report significant effects on body composition [16, 28]. On the contrary, a study indicated that L-carnitine can decrease weight, BMI, as well as waist and hip circumference [29]. Likewise, 2000 mg of L-carnitine along with hypo-caloric diet could reduce fat mass in diabetic patients [30]. The doses of L-carnitine supplementation in the mentioned study were two times higher than those used in our study, which may explain the discrepancy between the results. L-carnitine reduces weight and adipose tissue mass by oxidizing fat and decreasing the serum levels of leptin [31]. In addition, L-Carnitine intake may decrease BMI by increasing basal metabolism [32]. Moreover, obesity causes inflammation and lipid peroxidation by abnormal production of pro-inflammatory factors such as IL-6 and CRP as well as the release of free fatty acids from adipose tissue [4]. L-carnitine reduces activation of mitogen-activated protein kinases (MAPK). The MAPK induces expression of cytokines such as IL-6 [33]. In the present study, no significant differences were observed in CRP and MDA between the LCG and the PLG. In consistent with our results, 750 mg/d L-carnitine supplementation did not show any significant change on CRP and MDA in women with KOA [17, 18]. On the other hand, oral consumption of 1000 mg/d L-carnitine could significantly reduce CRP and MDA levels in patients with coronary arthritis disease [14, 15]. Likewise, propionil L-carnitine injection into hemodialysis patients improved the level of CRP [34]. Furthermore, a study on hemodialysis patients with hyper lipo-proteinemia reported that 1000 mg/d of oral L-carnitine could reduce inflammation but did not affect oxidative stress [19]. In comparison with the current research, CRP reduction in the mentioned studies may be due to the injection of L-carnitine and longer duration of the intervention.

Our findings showed that oral administration of L-carnitine did not lead to any significant improvement in lipid profile. Samimi et al. [29] showed that 12-week L-carnitine supplementation had no effect on lipid profile. In the same vein, a meta-analysis showed that L-carnitine supplementation could not improve TC, TG, and HDL-c in hemodialysis patients [13]. However, some studies reported that L-carnitine improved lipid profile in coronary artery disease and type 2 diabetes [35, 36]. This discrepancy can be due to the differences in dosage of L-carnitine. L-carnitine is a key cofactor in transferring fatty acids into mitochondria and causes incorporation of long-chain fatty acids into the β oxidation cycle to produce Acetyl-CoA. L-carnitine helps oxygen entrance into the tri-carboxylic acid (TCA) cycle to synthesize ATP and consequently decreases the concentration of oxygen and reduces formation of ROS [35].

The present study did not find any change in physical function and total score in WOMAC questionnaire. A study revealed that daily L-carnitine intake more than 2 g reduced stiffness, pain after prolonged movement, and disturbed sleep due to the pain [37]. Another study indicated that 750 mg oral L-carnitine supplementation significantly improves pain intensity and global assessment of disease status in patients with KOA [27]. Earlier studies suggest that L-carnitine enhances cell proliferation of cartilage matrix glycosaminoglycan component that resulted in inhibition of matrix degradation [10]. Weight loss reduces the mechanical pressure on the joints, which improves the score of the WOMAC scores [38]. It seems that longer duration interventions or higher doses of L-carnitine have a greater effect on weight and WC and consequent WOMAC scores.

The present study conducted the first clinical trial to compare the effect of L-carnitine supplementation along with low calorie diet on improving KOA in obese women by monitoring the diet and supplementation compliance. Moreover, L-carnitine appeared to be well tolerated by the participants. A limitation of the present study was that we did not evaluate the serum L-carnitine levels. Another important limitation of our study was the lack of measuring inflammatory markers, leptin, as well as synovial fluids of markers that are more related to obesity-mediated joint inflammation.

## Conclusion

According to our findings, oral administration of 1000 mg L-carnitine for 12 weeks could improve BMI, but had no significant impact on other anthropometric parameters, lipid profile, CRP, MDA, and WOMAC score.

## Abbreviations

BMI: Body mass index
CRP: C-reactive protein
HDL-c: High density lipoprotein-cholesterol
HC: Hip circumference
IQR: Inter quartile range
IPAQ: International Physical Activity Questionnaire
KOA: Knee Osteoarthritis
LCG: L-carnitine group
LDL-c: Low density lipoprotein cholesterol
MAPK: Mitogen-activated protein kinases
MDA: Malondialdehyde
MMP13: Matrix metallopeptidase13
NSAID: Nonsteroidal anti-inflammatory drug
OA: Osteoarthritis
PLG: Placebo group
ROS: Reactive oxygen species
TC: Total cholesterol
TCA: Tri-carboxylic acid
TG: Triglycerides
WC: Waist circumference
WOMAC: Western Ontario and McMaster Universities Osteoarthritis Index
